# Review of Analyses Estimating Relative Vaccine Effectiveness of Cell-Based Quadrivalent Influenza Vaccine in Three Consecutive US Influenza Seasons

**DOI:** 10.3390/vaccines10060896

**Published:** 2022-06-03

**Authors:** Constantina Boikos, Ian McGovern, Deborah Molrine, Justin R. Ortiz, Joan Puig-Barberà, Mendel Haag

**Affiliations:** 1 Seqirus Inc., Kirkland, QC H9H 4M7, Canada; constantina.boikos@seqirus.com; 2 Seqirus USA, Cambridge, MA 02139, USA; deborah.molrine@seqirus.com; 3 Center for Vaccine Development and Global Health, University of Maryland School of Medicine, Baltimore, MD 21201, USA; jortiz@som.umaryland.edu; 4 Foundation for the Promotion of Health and Biomedical Research (FISABIO), 46020 Valencia, Spain; jpuigb55@gmail.com; 5 Seqirus Inc., 1101 Amsterdam, CL, The Netherlands; mendel.haag@seqirus.com

**Keywords:** cell-based quadrivalent influenza vaccine, egg-based influenza vaccine, relative vaccine effectiveness, influenza

## Abstract

The adaptation of influenza seed viruses in egg culture can result in a variable antigenic vaccine match each season. The cell-based quadrivalent inactivated influenza vaccine (IIV4c) contains viruses grown in mammalian cell lines rather than eggs. IIV4c is not subject to egg-adaptive changes and therefore may offer improved protection relative to egg-based vaccines, depending on the degree of match with circulating influenza viruses. We summarize the relative vaccine effectiveness (rVE) of IIV4c versus egg-based quadrivalent influenza vaccines (IIV4e) to prevent influenza-related medical encounters (IRMEs) from three retrospective observational cohort studies conducted during the 2017–2018, 2018–2019, and 2019–2020 US influenza seasons using the same underlying electronic medical record dataset for all three seasons—with the addition of linked medical claims for the latter two seasons. We identified IRMEs using diagnostic codes specific to influenza disease (ICD J09*-J11*) from the records of over 10 million people. We estimated rVE using propensity score methods adjusting for age, sex, race, ethnicity, geographic location, week of vaccination, and health status. Subgroup analyses included specific age groups. IIV4c consistently had higher relative effectiveness than IIV4e across all seasons assessed, which were characterized by different dominant circulating strains and variable antigenic drift or egg adaptation.

## 1. Introduction

### 1.1. Egg Adaptation

Traditionally, influenza vaccines are manufactured in viral culture, a process in which an influenza vaccine seed virus must first bind to a cellular receptor to infect a cell. The receptors on the surface of avian cells differ from those on mammalian cells. For a human influenza virus to propagate efficiently in avian cells, it sometimes must adapt to bind the avian receptor; this process is known as egg adaptation. Importantly, adaption occurs in the region of the influenza virus that is dominant antigenically. Thus, as influenza viruses adapt to grow in eggs, antigenic differences from circulating viruses may arise. These differences may cause egg-based vaccines to be potentially less effective at preventing influenza virus infection than vaccines manufactured in other ways, including those with vaccine viruses grown in mammalian cell cultures or using recombinant technologies [1,2]. Although all influenza virus subtypes and lineages undergo mutational changes in egg culture, antigenic changes to the influenza A(H3N2) virus appear to be more frequent and profound [3,4].

### 1.2. Cell-Based Influenza Vaccine

The cell-based quadrivalent inactivated influenza vaccine (IIV4c; Flucelvax, Seqirus, Holly Springs, NC) contains viruses grown in a mammalian cell line. The cell-culture technology process isolates and grows viruses in Madin–Darby canine kidney (MDCK) cells rather than fertilized hen’s (or chicken) eggs, which are traditionally used for influenza vaccines. The production of cell-based influenza vaccines using cell-cultured candidate vaccine viruses (CVVs) does not appear to select for antigenic changes as does egg-propagation, and therefore permits the growth of viruses that are more similar to the expected circulating strain [1,5,6]. As such, IIV4c may offer improved protection relative to egg-based quadrivalent influenza vaccines (IIV4e). Beginning in the 2017–2018 season, IIV4c included an A(H3N2) cell seed strain. For the 2018–2019 season, A(H3N2) and both influenza B viruses were manufactured from cell-cultured seed strains. As of the Northern Hemisphere 2019–2020 influenza season, all four influenza strains in IIV4c are manufactured from cell-cultured seed strains.

### 1.3. Influenza Vaccine Effectiveness

Influenza vaccines are the best countermeasure against influenza virus infection. However, the effectiveness of current vaccines varies. Vaccine effectiveness is affected by many variables, including health status, age, prior influenza exposures of persons receiving the influenza vaccine, the outcome being prevented, and vaccine-specific qualities. The degree of vaccine match with circulating strains is a major factor affecting vaccine effectiveness. A vaccine mismatch occurs if the antigenic properties of circulating viruses differ significantly from those of the corresponding vaccine virus; an example would be an ≥8-fold reduction in the hemagglutination inhibition (HI) titer for circulating viruses of a given type or subtype compared with the homologous titer for the corresponding vaccine virus [7]. Antigenic drift occurs when viruses acquire mutations in hemagglutinin (HA) and neuraminidase (NA) antigenic sites in order to escape recognition by the host immune system. Egg adaptation represents another mechanism of vaccine mismatch that may occur during vaccine production. The occurrence of antigenic drift or egg-adaptation can variably impact influenza vaccine effectiveness depending on the degree of antigenic dissimilarity between circulating and vaccine viruses and the predominance of those viruses. 

A 2016 systematic review determined that pooled effectiveness against symptomatic influenza caused by A(H3N2) in the general outpatient population was as low as 23% (95% CI: 2% to 40%) when there was a mismatch between the vaccine and circulating viruses [8]. Additionally, the lowest pooled vaccine effectiveness against illness caused by A(H3N2) was observed in individuals older than 60 years of age (24%; CI: −6% to 45%), a finding that may reflect immunosenescence, which contributes to decreased protection from influenza vaccines in older adults [8,9].

### 1.4. Real-World Evidence

Randomized controlled trials (RCT) and real-world evidence (RWE) are both necessary and complementary components in understanding the public health value of seasonal influenza vaccination. RCTs are designed to evaluate the efficacy and safety in well-defined and usually healthy study populations. The observational nature of RWE studies permits the assessment of vaccine effectiveness more frequently and in larger and more inclusive populations in the context of routine care [10,11]. Alongside RCT data, RWE is used by public health authorities such as the US Centers for Disease Control and Prevention (CDC), World Health Organization (WHO), and other agencies to guide policy decisions, clinical practice recommendations, public health messaging, and vaccine development [12,13,14,15,16,17,18,19,20,21].

In this review, we discuss the relative vaccine effectiveness (rVE) of IIV4c versus IIV4e in children and adults over three consecutive influenza seasons from 2017 to 2020, as well as the epidemiological characteristics of each season. The rVE for each season was estimated based on the same underlying EMR dataset for all three seasons, with the addition of linked claims for the latter two seasons [22,23,24,25,26]. While the RWE evaluations of the rVE of IIV4c versus IIV4e may be best done in the context of the entire available body of evidence, disentangling the relative contributions of study design and season-specific effects on variations in the observed rVE presents a complex challenge. The rVE of IIV4c versus IIV4e has already been evaluated using a systematic literature review and meta-analysis approach [27]. In this review, the use of the same methodologies and databases across each season allow for the evaluation of how the differences in the underlying epidemiological characteristics of the influenza seasons may have contributed to variation in the rVE of IIV4c versus IIV4e.

## 2. Methodology of Individual Studies

This is a review of three retrospective cohort studies that were conducted during the 2017–2018, 2018–2019, and 2019–2020 influenza seasons. The study of the 2017–2018 season used a dataset of electronic medical records (EMRs) from primary care and specialty clinics (Veradigm Health Insights Ambulatory database; Allscripts Touchworks and Allscripts PRO, Chicago, IL, USA and Practice Fusion, Inc., San Francisco, CA, USA). For the subsequent two seasons, this EMR dataset was linked to available pharmacy and medical claims (Komodo Healthcare Map, Komodo Health Inc., New York, NY, USA). The dataset for each retrospective cohort study included only de-identified clinical data that met Protected Health Information (PHI) data requirements and were certified for Health Insurance Portability and Accountability Act (HIPAA) compliance. The studies were conducted and reported in accordance with Good Pharmacoepidemiological Practice from the Declaration of Helsinki, which is applicable to local regulations, and the Reporting of Studies Conducted using Observational Routinely Collected Health Data (RECORD) [22,23,24,25,26,28].

Eligible subjects were ≥4 years of age, resided in the US, and had a record of vaccination with IIV4c or IIV4e as determined using current procedural terminology (CPT), code for vaccine administered (CVX), and/or national drug codes (NDC) (Table S1). The observation period during the 2017–2018 season was from 1 August 2017 through 30 March 2018 and subjects received the vaccine from 1 August 2017 through 28 February 2018 [22]. During the next season, subjects were vaccinated from 1 August 2018 through 28 February 2019 and the observation period was 1 August 2018 through 18 May 2019 [23]. In the 2019–2020 season, subjects were vaccinated from 1 August 2019 through 31 January 2020 and the observation period was 29 September 2019 through 7 March 2020—an early cut-off to avoid potential bias arising from the coronavirus disease 2019 (COVID-19) pandemic [25,26]. Overall inpatient or outpatient influenza-related medical encounters (IRMEs)—the primary outcome in all studies—were defined using the International Classification of Diseases (ICD)–9-CM and ICD-10-CM codes that correspond to the US Armed Forces Health Surveillance Center (AFHSC) Code Set B (Table S2) [29]. Subgroup analyses by age (4–17, 18–64, and ≥65 years) were also evaluated in each of the three studies.

In this review, we focus on findings from each season that were derived using the same procedures—a propensity score adjustment methodology—to facilitate comparisons. Propensity score adjustment methods were used as a sensitivity analysis for the first season and as the primary analysis for the second two studies. The method adjusted for predefined covariates, including age, sex, race, ethnicity, geographic region, vaccination week, and health status, using an inverse probability of treatment-weighted (IPTW) methodology. For the 2017–2018 season analysis, a conditional logistic regression model using propensity score (PS)–matched pairs was used and the propensity scores were calculated based on age, sex, race, geographic region, and the presence of each of the 17 comorbidity categories included in the Charlson comorbidity index (CCI) [22,30]. For both the 2018–2019 and 2019–2020 seasons, doubly robust models were used that involved a multivariable logistic model to calculate PS for IPTW adjustment, and then the same covariates were included in a multivariable logistic model. For the 2018–2019 season, covariates included age, sex, race, ethnicity, geographic region, week of vaccination, and the presence of 17 comorbidity categories in CCI. For the 2019–2020 season, covariates included age, sex, race, ethnicity, region, index week, frailty index, individual CCI comorbidities, number of outpatient visits in baseline, and number of inpatient admissions in baseline. We also review a 2018–2019 subgroup analysis that was conducted among subjects with conditions that put them at high risk of influenza complications, including chronic pulmonary diseases, heart disease, cerebrovascular disease, renal disease, diabetes, malignancy or metastatic solid tumors, HIV/AIDS, rheumatic disease, and liver disease; this analysis was published separately [24,31]. For all seasons, rVE was determined using the formula (% VE = 1 − OR_adjusted_) × 100. Additional details on the statistical methods can be found in the original publications [22,23,24,25,26].

To provide context about overall influenza vaccine performance each study year, we also review results of the absolute vaccine effectiveness (VE) from the US CDC as well as studies of antigenic match between vaccine and circulating strains from each year.

## 3. Relative Effectiveness of IIV4c vs. IIV4e between 2017 and 2020

### 3.1. Influenza Epidemiology and Vaccine Effectiveness

**Figure 1:**
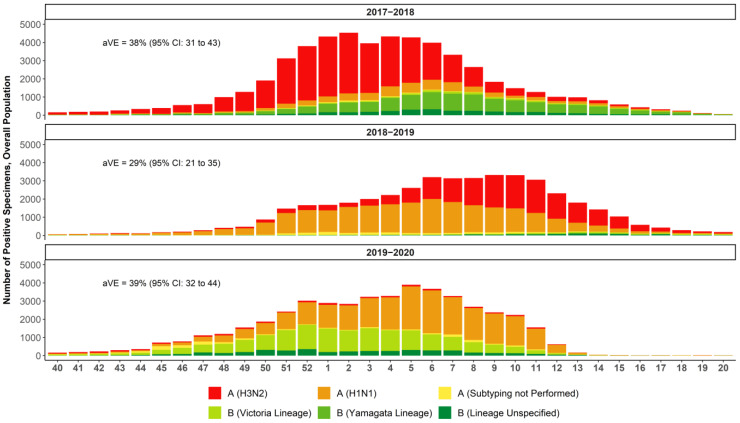
National summary of influenza-positive specimens as reported by public health laboratories in the US to the CDC and absolute vaccine effectiveness (aVE) as estimated by the CDC [32,38,42,43]. Week 40 is the last week in September/first week in October and week 20 ends mid-May.

The influenza seasons between 2017 and 2020 were characterized by different epidemiological patterns (Figure 1) [32]. To contextualize the observed rVE results, US CDC data on the severity of the season, burden of influenza estimates, strain circulation, and vaccine effectiveness data were reviewed and summarized. The CDC classifies season severity based on a combination of factors including influenza-like activity, rates of influenza hospitalizations, and the percent of deaths due to influenza and pneumonia in an influenza season [33]. The CDC uses modeling to estimate the number of influenza illnesses, medical visits, hospitalizations, and deaths in a season [34]. Strain circulation estimates are based on influenza subtyping performed by sentinel public health laboratories [35]. The CDC Flu VE Network estimates the overall vaccine effectiveness against medical visits due to laboratory-confirmed influenza [36].

**Figure 2:**
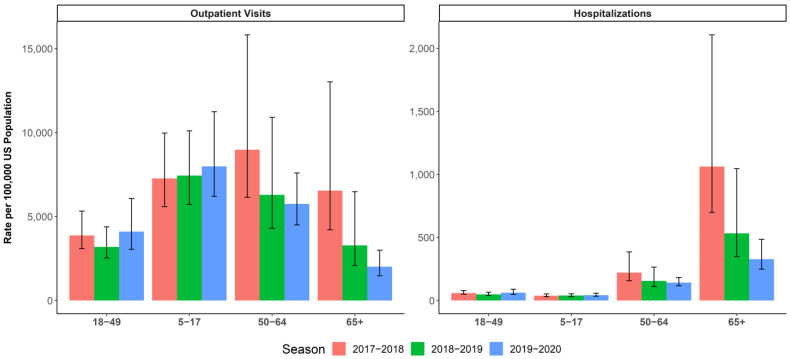
CDC-estimated burden of influenza (and 95% uncertainty interval) for the selected age groups (5–17, 18–49, 50–64, and ≥65 years) in season 2017–2018, 2018–2019, and 2019–2020 [37,40,41,44,45]. Left panel: rate of outpatient visits for influenza per 100,000. Right panel: rate of influenza hospitalizations per 100,000.

The 2017–2018 season was a high-severity season in which the most prevalent strain was A(H3N2), with some B Yamagata circulation in the latter half of the season and documented egg-adaptation for A(H3N2) [2,37,38,39]. The US CDC estimate of the adjusted absolute VE against medical visits due to laboratory-confirmed influenza caused by any influenza virus was 38% (95% CI: 31% to 43%) and 22% (95% CI: 12% to 31%) against the predominant A(H3N2) strain [38]. The 2018–2019 season was of moderate severity and marked by two waves in which A(H1N1) dominated in the first half of the season and A(H3N2) emerged in the second half [40,41]. The US CDC estimate of the overall adjusted absolute VE against medical visits due to laboratory-confirmed influenza was 29% (95% CI: 21% to 35%) and 9% (95% CI: −4% to 20%) against medical visits due to laboratory-confirmed influenza caused by A(H3N2) [42]. The 2019–2020 season was also considered to be of moderate severity, with higher infection rates than the 2018–2019 season but lower mortality and hospitalizations—except in persons 18–49 years of age (Figure 2) [41]. The first half of the season was marked by a wave of B Victoria, which tapered off as A(H1N1) began to dominate. Influenza virus infections during this season decreased precipitously as COVID-19 mitigation efforts began in the US in mid-March 2020; the CDC estimated that the overall adjusted absolute VE was 39% (95% CI: 32% to 44%) [32,43] against symptomatic influenza.

### 3.2. Relative Vaccine Effectiveness of IIV4c vs. IIV4e

A total of 1,353,862 vaccinated individuals were included in the analysis of the 2017–2018 season; 10,126,333 individuals in the 2018–2019 season; and 5,625,478 individuals in the analysis of the 2019–2020 season (Table 1). IIV4c recipients represented 7% of the study population in the first season, 21% in the second, and 27% in the third season. Changes in the total number of included individuals per season are likely multifactorial and may result from changes in vaccine market share by type, the addition of pharmacy claims for seasons two and three, changes in the underlying network of data providers captured by the database across seasons, the additional requirement of claims and EHR activity during the previous 12 months for the 2019–2020 season (as opposed to only the EHR activity in the previous two seasons), and the truncated vaccination period for the 2019–2020 season due to the COVID-19 pandemic. The most common demographic characteristics of the study populations (individual characteristics had frequency of >50%) included female sex, ethnicity/race of non-Hispanic/white, and residence in the southern US. Additional demographic details can be found in the original publications [22,23,25,26].

**Figure 3:**
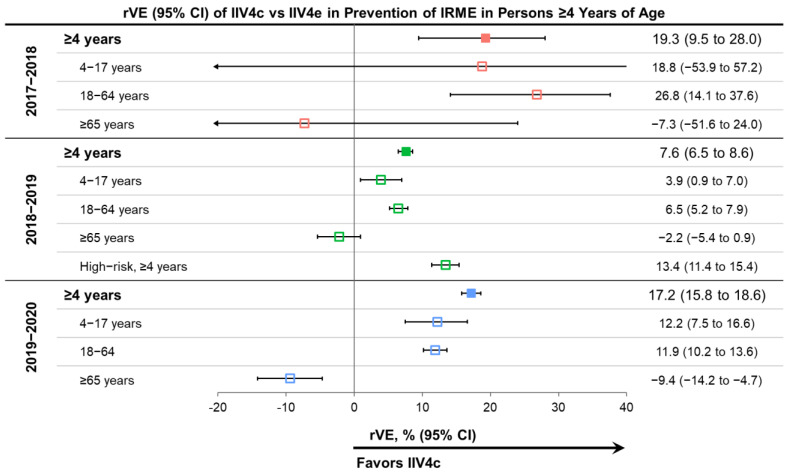
Relative vaccine effectiveness (rVE) of cell-based quadrivalent inactivated influenza vaccine (IIV4c) vs. egg-based quadrivalent inactivated influenza vaccine (IIV4e) during three influenza seasons between 2017 and 2020 in subjects ≥4 years of age and selected age subgroups [22,23,25,26]. Point estimates are color coded based on the season of the study indicated on the left side of the table. CI, confidence interval.

IIV4c performed better than IIV4e in the prevention of IRMEs in the overall population and in the pediatric and adult subgroups over the three consecutive US influenza seasons, except for the 4–17-year age group in 2017–2018 and adults ≥65 years old in all seasons. The rVE for the overall population ranged from 7.6% for the 2018–2019 season to 19.2% for the 2017–2018 season. IIV4c significantly improved protection from IRME relative to IIV4e in the overall population and the 18–64 age subgroup during all three seasons (Figure 3) [22,23,25,26]. In the pediatric population (4–17 years of age), the point estimates favored IIV4c over IIV4e in all seasons, but the effect was only statistically significant during the 2018–2019 and 2019–2020 seasons. The small number of pediatric IIV4c recipients (7465 vs. 404,510 IIV4e recipients) during the 2017–2018 season may have contributed to the wide confidence interval for the pediatric subgroup estimate for that season. 

The relative benefit of IIV4c over IIV4e was most prominent in the 2017–2018 season, during which A(H3N2) viruses predominated and there was also documented egg-adaptation in the A(H3N2) strains of IIV4e vaccines [2,39]. Point estimates of rVE were lower in the 2018–2019 season than the other two seasons but remained statistically significant across age groups—except for older adults—for IIV4c versus IIV4e. During the 2018–2019 season, the predominating A(H1N1) and A(H3N2) viruses were antigenically drifted from the vaccine virus and the A(H3N2) vaccine virus was probably affected by egg-adaptation [40,42,46,47]. The effect of egg-adaptation and drift was demonstrated by the absence of significant absolute VE against A(H3N2) viruses—with a CDC-estimated VE of 9% (95% CI: −4% to 20%) in 2018–2019 [42]. The impact of antigenic drift during the season may explain the attenuated rVE of IIV4c versus IIV4e during 2018–2019 compared to the other seasons. In the 2019–2020 season, the A(H1N1) virus in the vaccine was antigenically similar to the predominant circulating A(H1N1) virus based on antigenic characterization with ferret antisera [48,49]. However, in human serology studies, circulating A(H1N1) viruses had a decreased antigenic similarity to the cell-propagated reference virus and even more pronounced differences compared to an egg-propagated reference virus [48,49]. The human serology data suggest potential egg adaptation in combination with drift.

In the oldest age group (≥65 years old) during the 2017–2018 and 2018–2019 seasons, there were no significant differences between IIV4c and IIV4e. In 2019–2020, the rVE showed a benefit of IIV4e over IIV4c among patients aged ≥65 years old for protection from IRME relative to IIV4e. 

### 3.3. Effectiveness of IIV4c during Peak Influenza Activity and Impact of COVID-19

**Figure 4:**
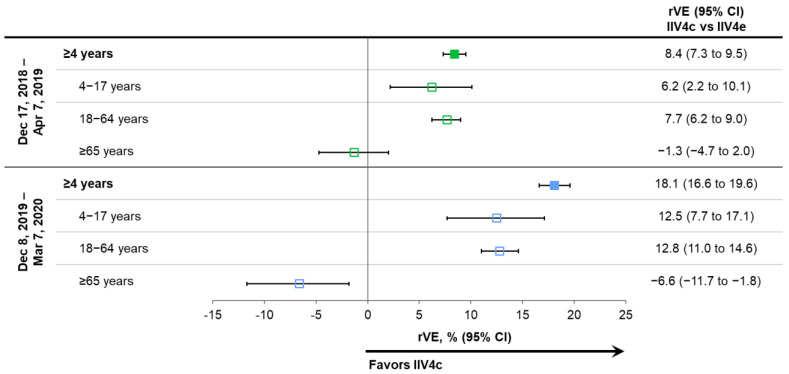
Relative vaccine effectiveness (rVE) of cell-based quadrivalent inactivated influenza vaccine (IIV4c) vs. egg-based quadrivalent inactivated influenza vaccine (IIV4e) during periods of peak influenza activity overall and in age subgroups for seasons 2018–2019 and 2019–2020 [23,25,26]. Point estimates are color coded based on the season of the study indicated on the left side of the table. CI, confidence interval.

As shown in Figure 4, the benefits of IIV4c relative to IIV4e remained consistent during periods of peak laboratory-confirmed influenza, which were analyzed in the latter two seasons (17 December 2018 through 7 April 2019 and 8 December 2019 through 7 March 2020) [23,25,26,32]. Note that this sensitivity analysis was not conducted for the 2017–2018 influenza season.

Influenza activity and/or healthcare-seeking behavior changed in mid-March 2020 (Figure 1) as COVID-19 mitigation measures were instituted throughout the US. In a sensitivity analysis of the full 2019–2020 influenza season (29 September 2019 through 16 May 2020), the benefits of IIV4c versus IIV4e were similar to those shown by the primary and peak activity analyses, with rVEs of 16.8% (95% CI: 15.1% to 18.4%) in the overall population, 12.0% (95% CI: 7.3% to 16.4%) in subjects 4–17 years of age, 12.1% (95% CI: 10.3% to 13.8%) in those aged 18–64 years, and –8.8% (95% CI: –13.7% to –4.1%) in those ≥65 years old.

### 3.4. Protection in High Risk Populations

A subgroup analysis conducted during 2018–2019 included 2,113,216 subjects ≥4 years of age with at least one condition that put them at high risk of influenza complications (Table 1, risk conditions listed in Table S3) [24,31]. This stratification was also applied to those ≥65 years of age. In the overall high-risk subgroup (≥4 years of age), the rVE of IIV4c versus IIV4e was 13.4% (95% CI: 11.4% to 15.4%), demonstrating a protective effect in vulnerable patient populations regardless of age. A significant rVE for IIV4c versus IIV4e was specifically seen for subjects with any chronic pulmonary disease (18.7% [95% CI: 16.0% to 21.3%]), asthma (21.4% [95% CI: 18.4% to 24.3%]), and rheumatic disease (11.8% [95% CI: 3.6% to 19.3%]) [24]. No differences were observed between IIV4c and IIV4e for subjects with myocardial infarction or chronic heart failure, cerebrovascular or peripheral vascular disease, renal disease, diabetes, any malignancy/metastatic tumors, HIV/AIDS, or liver disease. However, stratification by specific medical condition resulted in small subgroup sample sizes, limiting the statistical power to detect differences in vaccine effectiveness for many of the comparisons.

### 3.5. Hospitalizations and Outpatient Visits

During the 2019–2020 season, the CDC reported an estimated 74,717 influenza hospitalizations among adults 18–49 years of age and 86,171 influenza hospitalizations among adults 50–64 years of age [41]. In the overall population aged ≥4 years, IIV4c provided greater protection than IIV4e from influenza-related Hospitalizations with an influenza diagnosis in any diagnostic position, with an rVE of 5.7% (95% CI: 2.6% to 8.7%). Vaccination with IIV4c also resulted in a reduction in outpatient IRMEs compared to vaccination with IIVe for the 2019–2020 season, with rVEs of 20.8% (95% CI: 19.2% to 22.4%) in the overall population aged ≥4 years, 14.7% (95% CI: 12.7% to 16.7%) among adults 18–64 years of age, and 14.3% (95% CI: 9.3% to 19.0%) among children and adolescents [25,26].

## 4. Conclusions

Influenza causes significant morbidity and mortality globally. Vaccination against influenza is a central strategy in the prevention of influenza illness. However, vaccine effectiveness varies each season, with multiple factors contributing to sub-optimal vaccine effectiveness in some seasons. While many factors influence vaccine effectiveness, egg-adaptation related to the production of influenza vaccines in embryonated chicken eggs may be avoided by cell-culture technologies [3]. Cell-culture technology may improve the antigenic match between the vaccine virus reference strain and the vaccine selected strain and thus may improve vaccine effectiveness. This review summarizes trends in rVE estimates over three consecutive influenza seasons that were estimated using the same underlying US real world dataset originating from primary care (but expanded to integrated medical and pharmacy claims data for seasons two and three). Each study compared IIV4c to IIV4e in children and adults [22,23,24,25,26]. The results are discussed in the context of the epidemiological characteristics of the influenza viruses (strain circulation, burden, antigenic characterization) from surveillance data in each season. 

IIV4c often had higher relative effectiveness than IIV4e across three seasons from 2017 to 2020, which were characterized by different dominant circulating strains and different levels of drift or egg-adaptation. Currently, the US Advisory Committee on Immunization Practices (ACIP) does not preferentially recommend any specific vaccine over any other when more than one licensed, age-appropriate vaccine is available, particularly because all vaccine options are not uniformly available throughout the US [50]. However, consumers and clinicians may consider the relative vaccine performance data presented in this review when making choices about influenza vaccine products. The public health impact of improved relative vaccine effectiveness depends on the underlying absolute vaccine effectiveness of the two types of influenza vaccines evaluated (i.e., how well they protect against influenza compared to no vaccination) [51]. The studies included in this review do not provide absolute vaccine effectiveness estimates for the vaccines evaluated, but the provided CDC vaccine effectiveness estimates (which are not specific to any type of influenza vaccine) provide a general idea of how well influenza vaccines performed in a given season. 

For the ≥65 year age group, the rVE point estimates favored IIV4e over IIV4c in all three seasons evaluated, but the difference was only significant in the 2019–2020 season. The statistical methods used in the studies were intended to adjust for baseline characteristic differences between individuals who received one vaccine over another, which could be related to their risk of having influenza illness and/or seeking medical care for that illness. In the absence of a clear biological rationale for improved vaccine effectiveness of IIV4e versus IIV4c among older adults—given that both of these non-adjuvanted, inactivated vaccines have the same antigen content—it is possible our findings may be partially the result of residual confounding due to remaining patient characteristic imbalances between the two vaccine groups. A binary categorization of the presence/absence of certain CCI comorbidity groups may be sufficient for adjusting for health status differences for individuals younger than 65 years of age who tend to have fewer comorbidities, but it may be insufficient among older adults who may have multiple comorbidities. If having multiple comorbidities (or certain combinations of comorbidities) resulted in a multiplicative increase in the risk of influenza (i.e., a greater increased risk than the sum of the individual contributing comorbidities), then some degree of residual confounding may be expected.

The apparent lack of benefit of IIV4c versus IIV4e among patients aged ≥65 years of age suggests that other vaccines may be preferred for this population. Due to immunosenescence, vaccines enhanced with either the MF59^®^ adjuvant or a higher dose of influenza antigens have been designed for the population aged ≥65 years of age [52]. A recent systematic review of RWE concluded that, among adults ≥65 years of age, adjuvanted trivalent influenza vaccine was effective, with rVE estimates that favored the adjuvanted vaccine over nonadjuvanted, egg-based, standard-dose trivalent and quadrivalent influenza vaccines [53].

The reduction in IRMEs for the pediatric population and adults younger than 65 years of age observed in this study could do much to reduce the overall burden of influenza. Influenza infections in children and adults <65 years of age account for an estimated annual average economic burden of $1.84 billion in direct medical expenditures and $6.94 billion in indirect costs due to missed school by sick children and missed work by sick adults and the parents and guardians who care for children with influenza [54,55]. The results discussed in this review are part of a broader set of studies that have evaluated the relative vaccine effectiveness and cost effectiveness of IIV4c compared to IIV4e [27,56,57,58,59]. The use of RWE to assess rVE will remain an important source of data for the timely assessment of current and future seasons.

## Figures and Tables

**Table 1:** Numbers of vaccinated subjects with IIV4c or IIV4e included in the study population in each season.

	IIV4c				IIV4e			
	≥4 Years	4–17 Years	18–64 Years	≥65 Years	≥4 Years	4–17 Years	18–64 Years	≥65 Years
2017–2018	92,187	7465	55,104	29,618	1,261,675	404,510	693,014	164,151
2018–2019	2,125,430	78,602	1,529,189	517,639	8,000,903	1,628,038	5,384,922	987,943
2018–2019, high risk	471,301	—	—	—	1,641,915	—	—	—
2019–2020	1,499,215	60,480	1,144,427	354,788	4,126,263	1,240,990	3,427,818	698,445

## Data Availability

The datasets used in this study are privately licensed and are not available in order to maintain patient privacy.

## Supplementary Materials

The following are available online at https://www.mdpi.com/article/10.3390/vaccines10060896/s1, Table S1. List of CPT, CVX, and NDC codes used to identify influenza vaccines from the Veradigm EMR dataset, Table S2. Outcome case definitions, Table S3. ICD-9-CM and ICD-10 coding algorithms for Charlson comorbidities. Reference [60] is cited in the supplementary materials.
