# Supplementary material for: Review of Analyses Estimating Relative Vaccine Effectiveness of Cell-Based Quadrivalent Influenza Vaccine in Three Consecutive US Influenza Seasons
Source: Vaccines (Basel). 2022 Jun 3;10(6):896. doi: 10.3390/vaccines10060896 (PMC9228909; doi:10.3390/vaccines10060896)
Supplement: Supplementary file 1 [file vaccines-10-00896-s001.zip › vaccines-1658961-supplementary.pdf]

Supplemental Material

Table S1. List of CPT, CVX, and NDC codes used to identify influenza vaccines from the Veradigm EMR dataset.

**Influenza vaccine type**	**CPT**	**CVX**	**NDC**
IIV4c	90674, 90756	153,171,186	62577-0613-01, 62577-0614-01, 63851-0612-01, 63851-0613-01, 62577-0613-11, 62577-0614-11, 63851-0612-11, 63851-0613-11, 70461-0200-01, 70461-0200-11, 70461-0301-10, 70461-0418-10, 70461-0301-12, 70461-0418-11, 70461-0201-01, 70461-0318-03, 70461-0201-11, 70461-0318-04
IIV4e	90685, 90686, 90687, 90688	158,150,161	33332-0219-20, 33332-0316-01, 33332-0317-01, 33332-0318-01, 33332-0319-01, 33332-0416-10, 33332-0417-10, 33332-0418-10, 33332-0419-10, 33332-0219-21, 33332-0316-02, 33332-0317-02, 33332-0318-02, 33332-0319-02, 33332-0416-11, 33332-0417-11, 33332-0418-11, 33332-0419-11, 58160-0896-52, 58160-0900-52, 58160-0903-52, 58160-0905-52, 58160-0907-52, 58160-0898-52, 58160-0901-52, 58160-0896-41, 58160-0900-41, 58160-0903-41, 58160-0905-41, 58160-0907-41, 58160-0898-41, 58160-0901-41, 19515-0891-11, 19515-0894-52, 19515-0895-11, 19515-0897-11, 19515-0898-11, 19515-0901-52, 19515-0903-11, 19515-0906-52, 19515-0908-52, 19515-0896-11, 19515-0900-11, 19515-0909-52, 19515-0912-52, 19515-0891-01, 19515-0894-41, 19515-0895-01, 19515-0897-01, 19515-0898-01, 19515-0901-41, 19515-0903-01, 19515-0906-41, 19515-0908-41, 19515-0896-01, 19515-0900-01, 19515-0909-41, 19515-0912-41, 49281-0413-10, 49281-0413-50, 49281-0414-10, 49281-0414-50, 49281-0415-10, 49281-0416-10, 49281-0416-50, 49281-0417-10, 49281-0417-50, 49281-0418-10, 49281-0418-50, 49281-0419-10, 49281-0419-50, 49281-0513-25, 49281-0514-25, 49281-0516-25, 49281-0517-25, 49281-0518-25, 49281-0519-25, 49281-0621-15, 49281-0625-15, 49281-0627-15, 49281-0629-15, 49281-0631-15, 49281-0413-58, 49281-0413-88, 49281-0414-58, 49281-0414-88, 49281-0415-58, 49281-0416-58, 49281-0416-88, 49281-0417-58, 49281-0417-88, 49281-0418-58, 49281-0418-88, 49281-0419-58, 49281-0419-88, 49281-0513-00, 49281-0514-00, 49281-0516-00, 49281-0517-00, 49281-0518-00, 49281-0519-00, 49281-0621-78, 49281-0625-78, 49281-0627-78, 49281-0629-78, 49281-0631-78

CPT, current procedural terminology; CVX, code for vaccine administered; IIV4c, cell-based quadrivalent inactivated influenza vaccine; IIV4e, egg-derived quadrivalent inactivated influenza vaccines; NDC, national drug code.

Table S2. Outcome case definitions.

ICD-10-CM Codes	ICD-9-CM Codes
J09 (influenza due to certain identified influenza viruses)	—
J09.X (influenza due to identified novel influenza A viruses)	—
J09.X1 (influenza due to identified novel influenza A virus with pneumonia)	488.0 (influenza due to identified avian influenza virus)
	488.01 (influenza due to identified avian influenza virus with pneumonia)
	488.8 (influenza due to novel influenza A)<br>488.81 (influenza due novel influenza A with pneumonia)
J09.X2 (influenza due to identified novel influenza A virus with other respiratory manifestations)	488.02 (influenza due to identified avian influenza virus with other respiratory manifestations)
	488.82 (influenza due to novel influenza A with other respiratory manifestations)
J09.X3 (influenza due to identified novel influenza A virus with gastrointestinal manifestations)	488.09 (influenza due to identified avian influenza virus with other
	488.1 (influenza due to 2009 H1N1 influenza virus)
J09.X9 (influenza due to identified novel influenza A virus with other manifestations)	488.19 (influenza due to identified 2009 H1N1 influenza virus with other manifestations)
	488.89 (influenza due to novel influenza A with other manifestations)
J10 (influenza due to other identified influenza viruses)	—
J10.0 (influenza due to identified novel influenza A viruses)	—
J10.00 (influenza due to other identified influenza virus with unspecified type of pneumonia)	487.0 (influenza with pneumonia)
J10.01 (influenza due to other identified influenza virus with the same other identified influenza virus pneumonia)	487.1 (influenza with other respiratory manifestations)
J10.08 (influenza due to other identified influenza virus with other specified pneumonia)	487.0 (above)
	488.11 (influenza due to identified 2009 H1N1 influenza virus with pneumonia)
J10.1 (influenza due to other identified influenza virus with other respiratory manifestations)	487.1 (above)
	488.12 (influenza due to identified 2009 H1N1 influenza virus with other respiratory manifestations)
J10.2 (influenza due to other identified influenza virus with gastrointestinal manifestations)	487.8 (influenza with other manifestations)
J10.8 (influenza due to other identified influenza virus with other manifestations)	
J10.81 (influenza due to other identified influenza virus with other manifestations with encephalopathy)	
J10.82 (influenza due to other identified influenza virus with other manifestations with myocarditis)	

ICD-10-CM Codes	ICD-9-CM Codes
J10.83 (influenza due to other identified influenza virus with other manifestations with otitis media)	
J10.89 (influenza due to other identified influenza virus with other manifestations)	
J11 (influenza due to unidentified influenza virus)	—
J11.0 (influenza due to unidentified influenza virus with pneumonia)	—
J11.00 (influenza due to unidentified influenza virus with unspecified type of pneumonia)	487.0 (above)
J11.08 (influenza due to unidentified influenza virus with specified pneumonia)	
J11.1 (influenza due to unidentified influenza virus with other respiratory manifestations)	487.1 (above)
J11.2 (influenza due to unidentified influenza virus with gastrointestinal manifestations)	487.8 (above)
J11.8 (influenza due to unidentified influenza virus with other manifestations)	
J11.81 (influenza due to unidentified influenza virus with encephalopathy)	
J11.82 (influenza due to unidentified influenza virus with myocarditis)	
J11.83 (influenza due to unidentified influenza virus with otitis media)	
J11.89 (influenza due to unidentified influenza virus with other manifestations)	

Table S3. ICD-9-CM and ICD-10 coding algorithms for Charlson Comorbidities [1].

Charlson comorbidity category name	Deyo's ICD-9-CM +	ICD-10 *	Enhanced ICD-9-CM *
Myocardial infarction	410.x, 412.x	I21.x, I22.x, I25.2	410.x, 412.x
Congestive heart failure	428.x	I09.9, I11.0, I13.0, I13.2, I25.5, I42.0, I42.5-I42.9, I43.x, I50.x, P29.0	398.91, 402.01, 402.11, 402.91, 404.01, 404.03, 404.11, 404.13, 404.91, 404.93, 425.4-425.9, 428.x
Peripheral vascular disease	443.9, 441.x, 785.4, V43.4 Procedure 38.48	I70.x, I71.x, I73.1, I73.8, I73.9, I77.1, I79.0, I79.2, K55.1, K55.8, K55.9, Z95.8, Z95.9	093.0, 437.3, 440.x, 441.x, 443.1-443.9, 447.1, 557.1, 557.9, V43.4
Cerebrovascular disease	430.x-438.x	G45.x, G46.x, H34.0, I60.x-I69.x	362.34, 430.x-438.x
Dementia	290.x	F00.x-F03.x, F05.1, G30.x, G31.1	290.x, 294.1, 331.2
Chronic pulmonary disease	490.x-505.x, 506.4	I27.8, I27.9, J40.x-J47.x, J60.x-J67.x, J68.4, J70.1, J70.3	416.8, 416.9, 490.x-505.x, 506.4, 508.1, 508.8
Rheumatic disease	710.0, 710.1, 710.4, 714.0-714.2, 714.81, 725.x	M05.x, M06.x, M31.5, M32.x-M34.x, M35.1, M35.3, M36.0	446.5, 710.0-710.4, 714.0-714.2, 714.8, 725.x
Peptic ulcer disease	531.x-534.x	K25.x-K28.x	531.x-534.x
Mild liver disease	571.2, 571.4-571.6	B18.x, K70.0-K70.3, K70.9, K71.3-K71.5, K71.7, K73.x, K74.x, K76.0, K76.2-K76.4, K76.8, K76.9, Z94.4	070.22, 070.23, 070.32, 070.33, 070.44, 070.54, 070.6, 070.9, 570.x, 571.x, 573.3, 573.4, 573.8, 573.9, V42.7
Diabetes without chronic complication	250.0-250.3, 250.7	E10.0, E10.l, E10.6, E10.8, E10.9, E11.0, E11.1, E11.6, E11.8, E11.9, E12.0, E12.1, E12.6, E12.8, E12.9, E13.0, E13.1, E13.6, E13.8, E13.9, E14.0, E14.1, E14.6, E14.8, E14.9	250.0-250.3, 250.8, 250.9
Diabetes with chronic complication	250.4-250.6	E10.2-E10.5, E10.7, E11.2-E11.5, E11.7, E12.2-E12.5, E12.7, E13.2-E13.5, E13.7, E14.2-E14.5, E14.7	250.4-250.7
Hemiplegia or paraplegia	344.1, 342.x	G04.1, G11.4, G80.1, G80.2, G81.x, G82.x, G83.0-G83.4, G83.9	334.1, 342.x, 343.x, 344.0-344.6, 344.9
Renal disease	582.x, 583-583.7, 585.x, 586.x, 588.x	I12.0, I13.1, N03.2-N03.7, N05.2-N05.7, N18.x, N19.x, N25.0, Z49.0-Z49.2, Z94.0, Z99.2	403.01, 403.11, 403.91, 404.02, 404.03, 404.12, 404.13, 404.92, 404.93, 582.x, 583.0-583.7, 585.x, 586.x, 588.0, V42.0, V45.1, V56.x
Any malignancy, including lymphoma and	140.x-172.x, 174.x-195.8, 200.x-208.x	C00.x-C26.x, C30.x-C34.x, C37.x-C41.x, C43.x, C45.x-C58.x, C60.x-	140.x-172.x, 174.x-195.8, 200.x-208.x, 238.6

Charlson comorbidity category name	Deyo's ICD-9-CM +	ICD-10 *	Enhanced ICD-9-CM *
leukemia, except malignant neoplasm of skin		C76.x, C81.x-C85.x, C88.x, C90.x-C97.x	
Moderate or severe liver disease	456.0-456.21, 572.2-572.8	I85.0, I85.9, I86.4, I98.2, K70.4, K71.1, K72.1, K72.9, K76.5, K76.6, K76.7	456.0-456.2, 572.2-572.8
Metastatic solid tumor	196.x-199.1	C77.x-C80.x	196.x-199.x
AIDS/HIV	042.x-044.x	B20.x-B22.x, B24.x	042.x-044.x

Reference

1. Quan, H.; Sundararajan, V.; Halfon, P.; Fong, A.; Burnand, B.; Luthi, J.C.; Saunders, L.D.; Beck, C.A.; Feasby, T.E.; Ghali, W.A. Coding algorithms for defining comorbidities in ICD-9-CM and ICD-10 administrative data. *Med. Care* **2005**, *43*, 11.
